# An ADAM-10 dependent EPCR shedding links meningococcal interaction with endothelial cells to purpura fulminans

**DOI:** 10.1371/journal.ppat.1006981

**Published:** 2018-04-09

**Authors:** Hervé Lécuyer, Zoé Virion, Jean-Philippe Barnier, Soraya Matczak, Sandrine Bourdoulous, Elsa Bianchini, François Saller, Delphine Borgel, Xavier Nassif, Mathieu Coureuil

**Affiliations:** 1 Institut Necker Enfants Malades, INSERM U1151, CNRS UMR8253, Paris, France; 2 Université Paris Descartes, Paris, France; 3 Assistance Publique–Hôpitaux de Paris, Hôpital Universitaire Necker Enfants Malades, Service de Microbiologie Clinique, Paris, France; 4 Institut Cochin, INSERM U1016, CNRS UMR8104, Paris, France; 5 INSERM UMR-S1176, Université Paris-Sud, Université Paris Saclay, Le Kremlin-Bicêtre, France; 6 Assistance Publique–Hôpitaux de Paris, Hôpital Universitaire Necker Enfants Malades, Service d’Hématologie Biologique, Paris, France; Purdue University, UNITED STATES

## Abstract

*Purpura fulminans* is a deadly complication of *Neisseria meningitidis* infections due to extensive thrombosis of microvessels. Although a Disseminated Intra-vascular Coagulation syndrome (DIC) is frequently observed during Gram negative sepsis, it is rarely associated with extensive thrombosis like those observed during meningococcemia, suggesting that the meningococcus induces a specific dysregulation of coagulation. Another specific feature of *N*. *meningitidis* pathogenesis is its ability to colonize microvessels endothelial cells via type IV pili. Importantly, endothelial cells are key in controlling the coagulation cascade through the activation of the potent anticoagulant Protein C (PC) thanks to two endothelial cell receptors among which the Endothelial Protein C Receptor (EPCR). Considering that congenital or acquired deficiencies of PC are associated with *purpura fulminans*, we hypothesized that a defect in the activation of PC following meningococcal adhesion to microvessels is responsible for the thrombotic events observed during meningococcemia. Here we showed that the adhesion of *N*. *meningitidis* on endothelial cells results in a rapid and intense decrease of EPCR expression by inducing its cleavage in a process know as shedding. Using siRNA experiments and CRISPR/Cas9 genome edition we identified ADAM10 (A Disintegrin And Metalloproteinase-10) as the protease responsible for this shedding. Surprisingly, ADAM17, the only EPCR sheddase described so far, was not involved in this process. Finally, we showed that this ADAM10-mediated shedding of EPCR induced by the meningococcal interaction with endothelial cells was responsible for an impaired activation of Protein C. This work unveils for the first time a direct link between meningococcal adhesion to endothelial cells and a severe dysregulation of coagulation, and potentially identifies new therapeutic targets for meningococcal *purpura fulminans*.

## Author summary

*Neisseria meningitidis* (meningococcus) is responsible for a severe syndrome called *purpura fulminans* in which the coagulation system is totally dysregulated, leading to an extensive occlusion of blood microvessels. The pathogenesis of this syndrome is still not understood. Here we show that the meningococcus, when adhering on the apical surface of endothelial cells, induces the activation of membranous protease named ADAM-10, which in turn hydrolyses a cellular receptor called EPCR. The latter is key for the activation of a circulating potent anticoagulant, the Protein C (PC). PC activation is then impaired following meningococcal adhesion on endothelial cells. This work unveils for the first time a specific dysregulation of coagulation induced by the meningococcus and potentially identifies new therapeutic targets for meningococcal *purpura fulminans*.

## Introduction

*Neisseria meningitidis* is a natural host of the human nasopharynx. For still unknown reasons, it can invade the bloodstream, causing a severe disease with an annual incidence of about 1 case per 100 000 inhabitants in developed countries.

During meningococcemia, patients usually present cutaneous purpuric lesions [1]. These are the consequence of dermis microvessels thrombosis that induce capillary congestion and red blood cells extravasation. In about 25% of patients, these lesions evolve to an extended skin necrosis associated with a severe septic shock, a syndrome referred to as *purpura fulminans* (PF) [2–4]. Other organs can be affected by thrombosis and necrosis such as kidneys, heart and adrenal glands [5–7]. Despite highly active antimicrobials and intensive care, PF is still associated with a high mortality rate. Moreover, in surviving patients, PF lesions often require surgical debridement and limb amputations [8,9]. Deciphering PF pathogenesis is then a real need to develop new specific therapeutics to limit extensive thrombosis.

As for a Gram-negative sepsis, a Disseminated Intravascular Coagulation (DIC) syndrome is commonly observed during meningococcemia. This complication is due to high levels of circulating endotoxin and pro-inflammatory cytokines which trigger the coagulation cascade that ends with thrombin activation and subsequent fibrinogen cleavage. However, PF remains very unusual in Gram negative sepsis, whereas meningococcemia is usually complicated by mild to severe thrombotic events. This suggests that an additional dysregulation of coagulation occurs during this specific infection. Interestingly, apart from meningococcemia, PF also arise from any severe acquired or congenital deficit in the anti-coagulant protein C (PC). PC is a non-active zymogen produced by the liver that is activated by the endothelial cells following the generation of thrombin by the coagulation cascade. Thrombin bound on thrombomodulin, located on the surface of endothelial cells, cleaves PC in activated PC (aPC). Activated PC subsequently inactivates the coagulation cascade factors V and VIII, creating an endothelial-based negative feed-back on coagulation activation. The fixation of PC on the Endothelial Protein C Receptor (EPCR) accelerates the rate of aPC generation and has notably been proven to be critical during sepsis [10–14]. Importantly, a reduced endothelial expression of EPCR has been described at the site of meningococcal purpuric lesions [15]. This reflects a local impairment of PC activation which is likely to favor the formation of thrombosis. However, the molecular mechanism involved in this localized reduction of the EPCR endothelial expression remained unknown.

In addition to its pro-thrombotic nature, another specificity of meningococcemia is the ability of the bacteria to adhere on endothelial cells and to colonize microvessels [16–19]. In particular, this endothelial colonization has been demonstrated in skin biopsies from purpuric lesions [17,19,20]. This adhesion is the consequence of the interaction of the bacterial type IV pilus (T4P) with its cellular receptor CD147 [21]. T4P is a fiber composed of a major pilin PilE and of minor pilins designated as PilV, PilX and ComP [22]. Alongside its role in bacterial adhesion, T4P also triggers endothelial cells signaling. For instance, the T4P-induced a biased activation of the β2-adrenergic receptor that weakens the endothelial tight junctions and, at the level of the blood-brain barrier, opens the paracellular route to the meninges [23–25].

In this work, we hypothesized that the meningococcal interaction with endothelial cells is linked to the decrease of EPCR expression described at the site of purpuric lesions, leading to a subsequent impairment of PC activation, which is known to be key in thrombosis development. Using an *in vitro* approach, we demonstrate that meningococcus induces an EPCR shedding as a consequence of T4P-dependent adhesion. We also demonstrate that ADAM-10 (A Disintegrin and Metalloprotease 10) is the membranous protease responsible for this EPCR shedding, identifying for the first time a new sheddase for this receptor. Finally, we show that this ADAM10-dependant EPCR shedding induced by meningoccal interaction with endothelial cell is responsible for an impairment of PC activation.

## Results

### *N*. *meningitidis* T4P-dependent interaction with endothelial cells induces a rapid and important decrease of EPCR expression

As mentioned above, EPCR endothelial expression is reduced in purpuric lesions observed in children suffering from severe meningococcemia [15]. These cutaneous lesions correspond to location where bacteria colonize the microvessels [17,19], suggesting that the reduced expression of EPCR is the consequence of the meningococcal-endothelial cell interaction.

**Fig 1:**
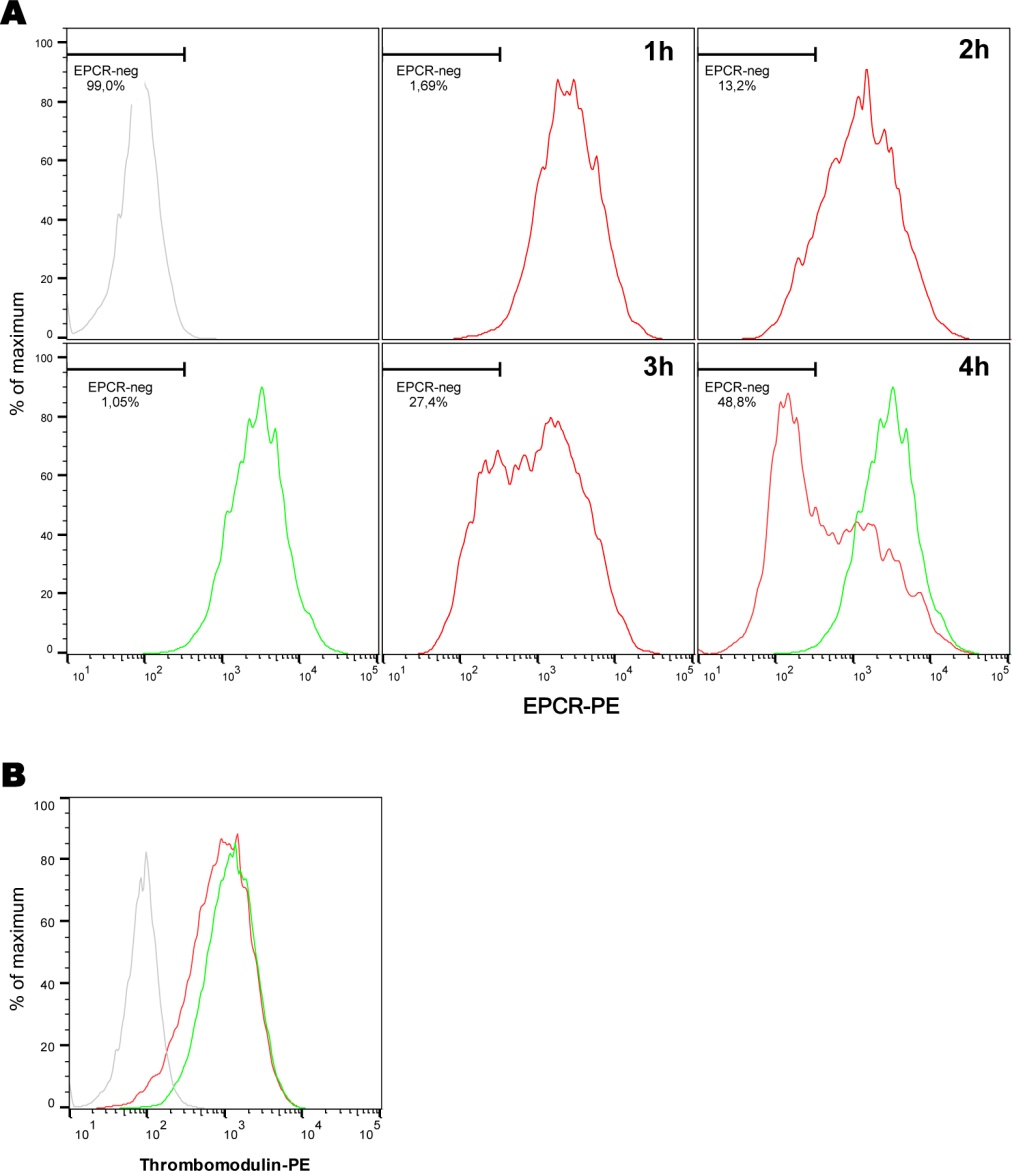
Expression of EPCR and thrombomodulin by endothelial cells after infection by *N*. *meningitidis*. Human Dermal Microvessels Endothelial Cells (HDMEC) were infected with a wild-type meningococcus (red) or left non-infected (green). Grey: isotype control. EPCR or thrombomodulin membranous expression were assessed by a FACS analysis. **(A)** Time course of EPCR expression during 4 hours of infection. The % of cells with no EPCR staining (EPCR-neg) is also indicated. EPCR-neg population was gated using non-infected cells. For the quantification of EPCR expression see supplemental data **S1 Fig**. **(B)** Thrombomodulin expression after 4 hours of infection. Data are representative of 3 independent experiments.

To test this hypothesis, we infected primary Human Dermal Microvessels Endothelial Cells (HDMEC) with the adhesive piliated wild type (WT) meningococcal strain 2C4.3 and assessed the membranous expression of EPCR using a FACS analysis without cell permeabilization. As shown **Fig 1A**, the infection induced a rapid and dramatic reduction of EPCR membranous expression. This started as early as 2 hours after the infection of the monolayer. On the other hand, 4 hours of infection had no effect on thrombomodulin expression which remained identical to that of non-infected cells (**Fig 1B**).

**Fig 2:**
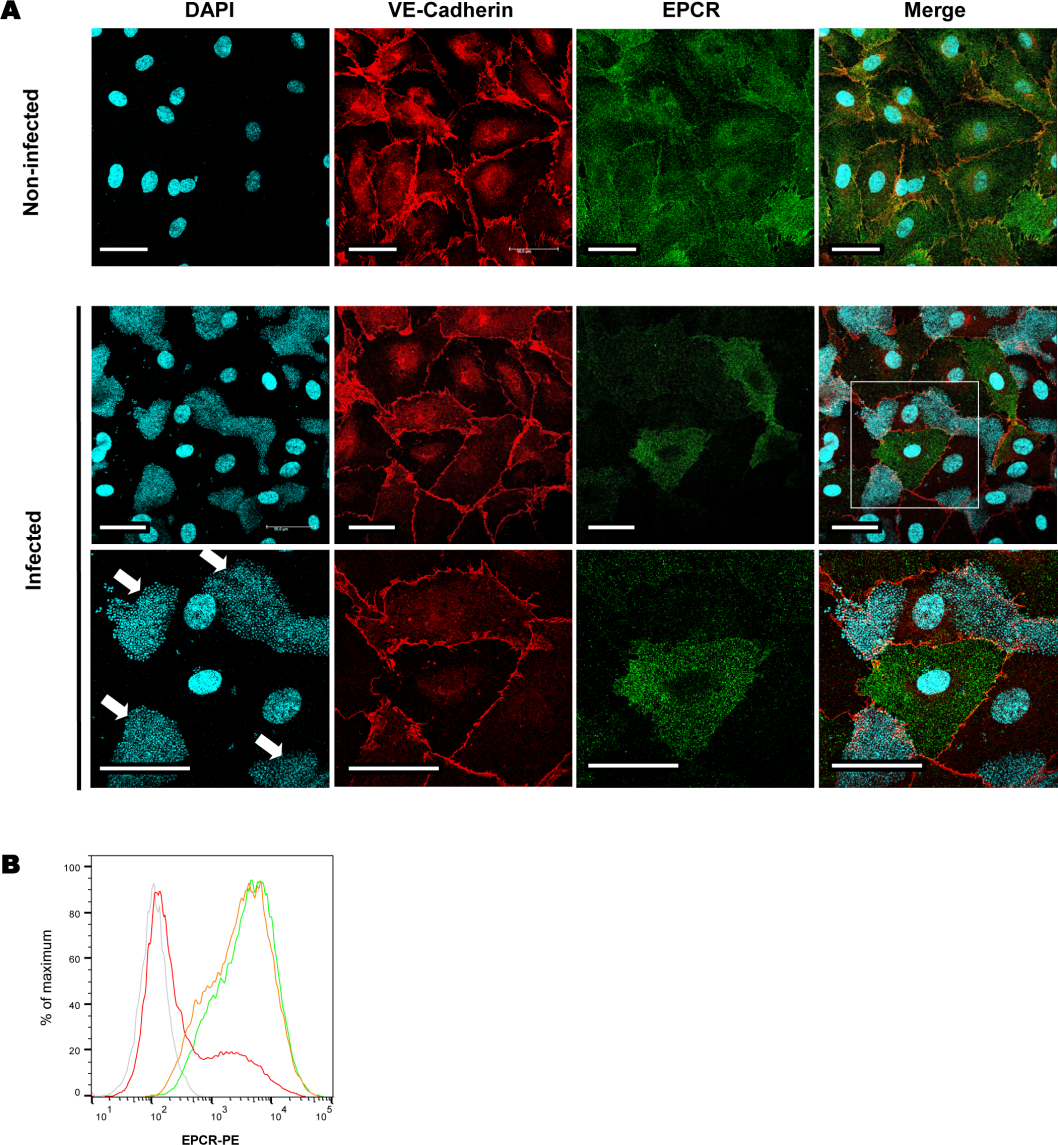
Meningococcal adhesion on endothelial cells is required to decrease EPCR expression. **(A)** HDMEC cells were left non-infected or were infected for 4h with a WT strain. DNA (cyan), junctionnal VE-Cadherin (red), or membranous EPCR (green) were stained using appropriate antibodies or DAPI. Bacteria were also stained using DAPI. Upper panel: non-infected cells. Medium panel: infected cells. Lower panel is a detailed view of the infected monolayer showing a non-infected cell surrounded by cells with *N*. *meningitidis* microcolonies (arrows) on the apical side. Scale bar: 50 μM. **(B)** HDMEC cells were infected for 4 hours with a WT piliated *N*. *meningitidis* strain (red) or a non-adherent non-piliated Δ*pilE* strain (orange) or left non-infected (green). EPCR expression was assessed by a FACS analysis. Data are representative of 3 independent experiments. (grey: isotype control). For the quantification of EPCR expression see supplemental data **S1 Fig**.

We then aimed at determining if this decrease in EPCR expression was a direct consequence of meningococcus adhesion onto endothelial cells. As shown **Fig 2A**, after 4 hours of infection the expression of membranous EPCR was dramatically reduced on colonized cells, while neighbouring non-colonized cells still expressed this receptor. Furthermore, the infection of a cell monolayer with a non-adhesive non-piliated isogenic derivatives (Δ*pilE*) had no effect on EPCR expression which remained identical to that of non-infected cells (**Fig 2B)**. Altogether these results reveal a direct link between meningococcal adhesion on endothelial cells and the decrease of EPCR membranous expression.

**Fig 3:**
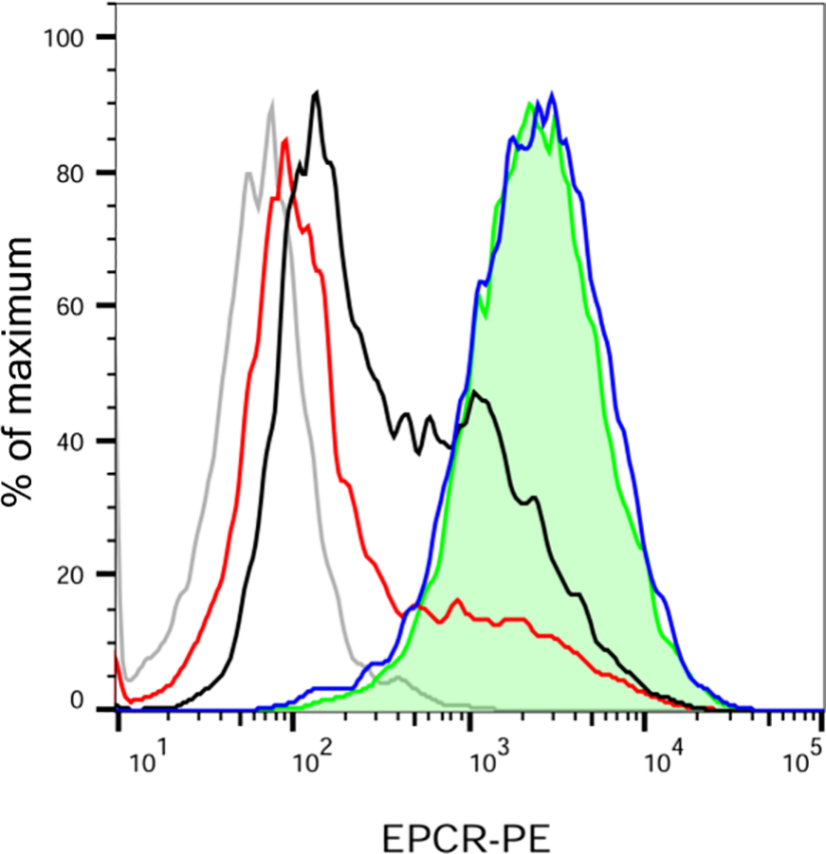
Meningococcal type IV pili trigger the decrease of EPCR expression. HDMEC were infected with either a WT *N*. *meningitidis* strain at a Multiplicity Of Infection (MOI) = 25 (red), Opa+Δ*SiaD* strain at MOI = 25 (black), Opa+Δ*SiaD*Δ*pilE* strain at MOI = 500 (blue–see Material and Methods) or left uninfected (green, tinted) for 4 hours (grey: isotype control). EPCR expression was assessed by a FACS analysis. Data are representative of 3 independent experiments. (grey: isotype control). For the quantification of EPCR expression see supplemental data **S1 Fig**.

As already mentioned the mean by which virulent capsulated meningococci interact with endothelial cells is via their T4P. To demonstrate that the reduction of endothelial EPCR expression was a specific consequence of T4P-mediated adhesion, we used a previously described isogenic derivative of the WT strain which is non-piliated (Δ*pilE)* and non-capsulated (Δ*siaD)* but expresses Opa adhesins that enable its adhesion on endothelial cells (Opa+Δ*pilE*Δ*siaD* strain—see Material and Methods). An isogenic piliated non-capsulated Opa-expressing strain (Opa+Δ*siaD*) was used as a control to rule out a possible role of the capsule. As shown **Fig 3**, EPCR expression after infection with the Opa+Δ*pilE*Δ*siaD* strain was comparable to that of non-infected cells. On the other hand, both the WT and the piliated non-capsulated strain (Opa+Δ*siaD*) induced a decrease of EPCR expression.

Altogether these data demonstrate that meningococcus T4P-dependent adhesion is responsible for a rapid and intense reduction of EPCR expression.

### Meningococcus interaction with endothelial cells induces the shedding of EPCR

**Fig 4:**
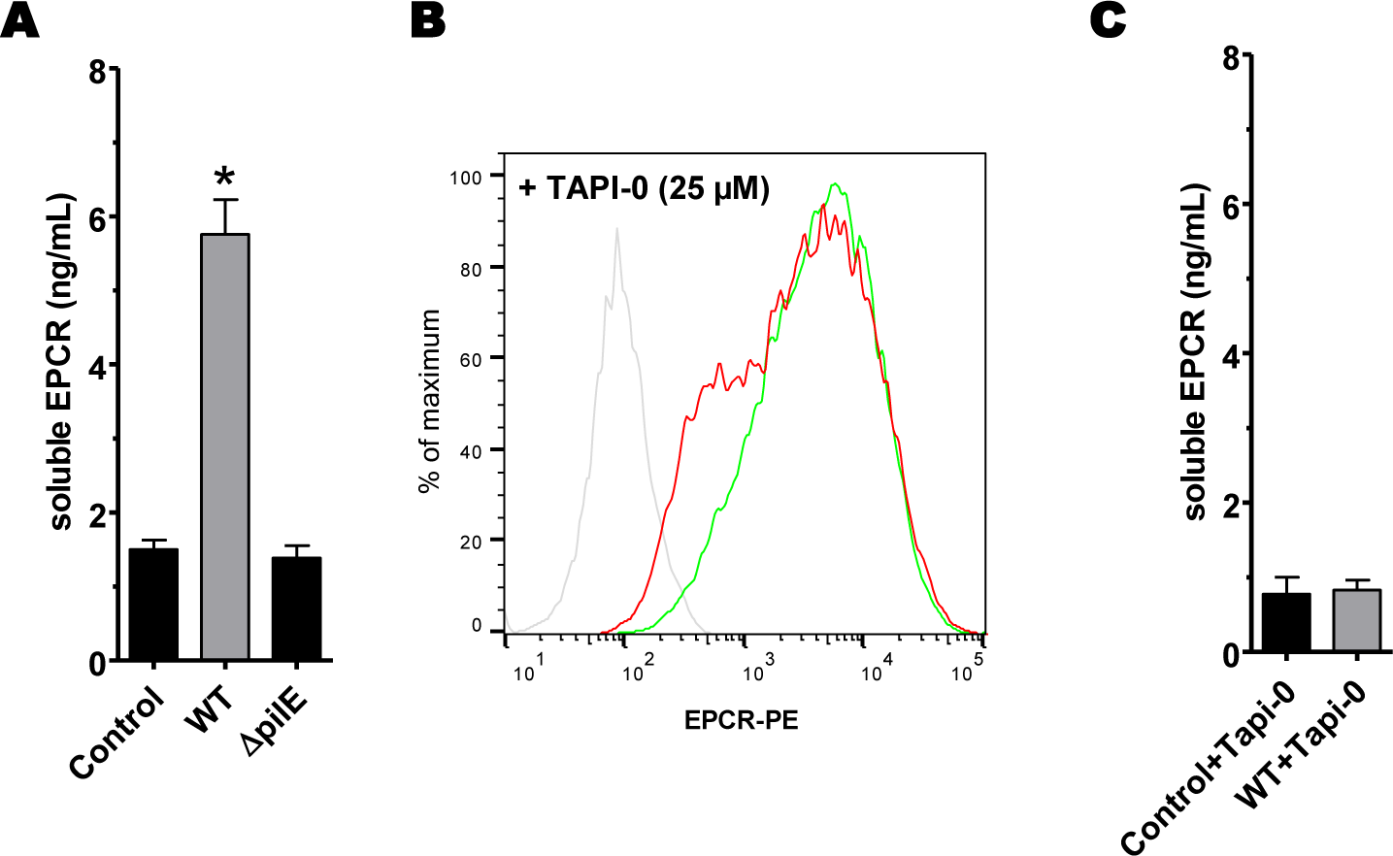
Meningococcal adhesion on endothelial cells induces an ADAM-dependant shedding of EPCR. **(A)** HDMEC were infected for 4 hours with a WT strain, a non-adherent non-piliated Δ*pilE* strain, or left non-infected (control). The amount of soluble forms of EPCR (sEPCR) was determined in the supernatant of cells by ELISA after infection. Data are mean +/- SEM of 3 independent experiments. * *p* = 0.0001 versus control (t-test). **(B)** HDMEC cells were treated for 1 hour with Tapi-0 (25μM), a pan-ADAM inhibitor, then infected with a WT strain (red) or left non-infected (green). EPCR expression was assessed by a FACS analysis. Data are representative of at least 3 independent experiments. (grey: isotype control). For the quantification of EPCR expression see supplemental data **S1 Fig**. **(C)** Amount of soluble EPCR in the supernatant determined by ELISA. Data are mean +/- SEM of 3 independent experiments.

As shown **Fig 1A**, the decrease of EPCR expression starts as early as 2 hours after infection of an endothelial cells monolayer. Such a rapid decrease of membranous EPCR suggested that the protein has been proteolytically removed from the cellular membrane. EPCR is indeed known to be a target of ADAM-17, a membranous protease that, upon its activation, cleaves the extracellular domain of the receptor [26]. To confirm the shedding hypothesis of the EPCR, we quantified the presence of soluble forms resulting from the cleavage of the receptor (sEPCR) in the supernatant of HDMEC cells infected for 4 hours with either a WT or a non piliated (Δ*pilE*) strain. As shown **Fig 4A**, sEPCR concentration in the supernatant of WT-infected cells was increased when compared to that of non-infected or Δ*pilE*-infected cells. To further support this result, we studied the membranous expression of EPCR after 4 hours of infection with a WT strain in the presence of 25 μM of TAPI-0, a drug known to inhibit all metalloproteases of the ADAM family, including ADAM17. We first checked that this drug had no effect on *N*. *meningitidis* growth and adhesion on endothelial cells (**S2 Fig**). As shown **Fig 4B**, membranous expression of EPCR after infection in the presence of 25 μM of TAPI-0 was almost identical to that of non-infected cells. Moreover, TAPI-0 treatment inhibited the release of soluble forms of EPCR in the supernatant of WT-infected cells (**Fig 4C**).

Altogether these data demonstrate that meningococcal interaction with endothelial cells induces the shedding of EPCR most likely via the activation of an ADAM-family protease.

### Meningococcus-induced EPCR shedding is ADAM10-dependent

**Fig 5:**
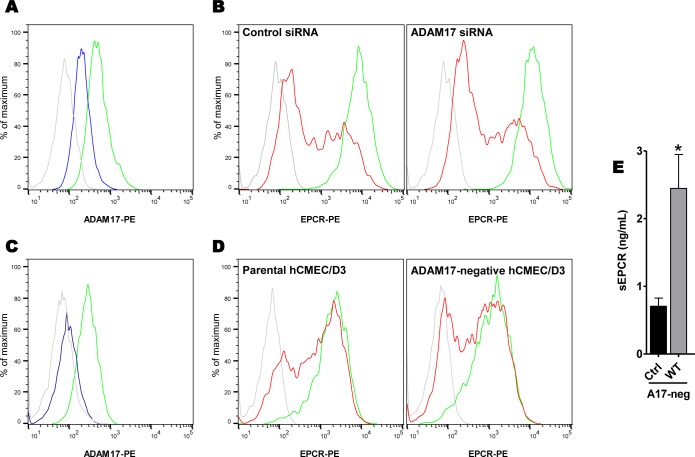
ADAM17 is not responsible for the meningococcus-induced EPCR shedding. **(A)** ADAM17 membranous expression of HDMEC cells transfected with a siRNA against ADAM17 (blue) or control (green). Grey: isotype control. For the quantification of ADAM17 expression see supplemental data **S3 Fig. (B)** EPCR expression in HDMEC cells transfected with either a control siRNA (left) or ADAM17 siRNA (right) and infected with a WT *N*. *meningitidis* strain for 4 hours (red) or left non-infected (green). Grey: isotype control. For the quantification of EPCR expression see supplemental data **S3 Fig.** A Crispr/Cas9-mediated knockdown of ADAM17 expression was performed as described in Material and Methods in the Human Cerebral Microvessels Endothelial Cells line hCMEC/D3. **(C)** ADAM17 expression in the resulting cell line (blue) compared to that of the parental cell line (green). **(D)** EPCR expression in parental hCMEC/D3 cell line (left) or ADAM17-negative-hCMEC/D3 cell line (right) infected with a WT *N*. *meningitidis* strain for 4 hours (red) or left non-infected (green). The adhesion of *N*. *meningitidis* on hCMEC/D3 is reduced compared to the adhesion on HDMEC explaining the slighty reduced shedding phenotype observed with hCMEC/D3. Data are representative of 3 independent experiments. (grey: isotype control). For the quantification of EPCR expression see supplemental data **S5 Fig. (E)** Soluble EPCR in the supernatant of ADAM17-negative-hCMEC/D3 cells after infection with a WT strain or non-infected (control) cells. Data are mean (+/- SEM) of 3 independent experiments. *:*p* = 0.007 (t-test).

We showed that an inhibitor of the whole ADAM family prevented the meningococcal-induced EPCR shedding. To date, the only *sheddase* described as being able to cleave the EPCR is ADAM17 [26]. To investigate the role of ADAM17 in the *N*. *meningitidis*-induced EPCR shedding, we first inhibited the expression of ADAM17 by transfecting HDMEC cells with a small interfering RNA (siRNA) against ADAM17 or a control siRNA. As shown **Fig 5A**, ADAM17 expression was reduced by ~70% in ADAM17-siRNA-transfected cells (blue) when compared to that of control-siRNA-transfected cells (green). Yet, EPCR shedding induced by *N*. *meningitidis* 4 hours after infection was identical in ADAM17-siRNA or control-siRNA transfected cells (**Fig 5B**). To confirm this result, we engineered an ADAM17-negative endothelial cell line using a Crispr/Cas9 technology in the human endothelial cerebral microvessel cell line hCMEC/D3 as described in the material and methods section. The resulting cell line was indeed ADAM17-negative as assessed by a FACS analysis (**Fig 5C**). The infection of these cells by a WT meningococcus strain induced a strong decrease in the expression of EPCR comparable to that observed with the parental hCMEC/D3 control cells (**Fig 5D**). Moreover, soluble forms of EPCR were still released upon infection (**Fig 5E**). These data clearly demonstrate that meningococcus-induced shedding of ECPR is ADAM17-independent.

**Fig 6:**
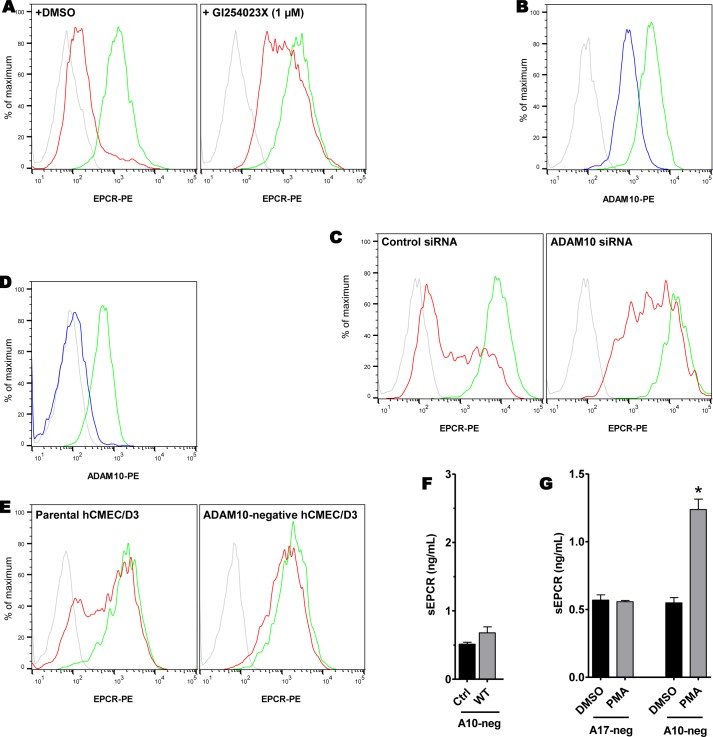
The EPCR shedding induced by meningococcus is mediated by ADAM10. (**A**) HDMEC were treated with the ADAM10 inhibitor GI254023X (1μM, right), or DMSO only (left) and were infected for 4h by a wild-type adherent *N*. *meningitidis* (red) or left non-infected (green). EPCR expression was assessed by a FACS analysis (grey: isotype control). Data are representative of 3 independent experiments. For the quantification of EPCR expression see supplemental data **S1 Fig**. **(B)** ADAM10 membranous expression of HDMEC cells transfected with a siRNA against ADAM10 (blue) or control (green). Grey: isotype control. For the quantification of ADAM10 expression see supplemental data **S3 Fig**. **(C)** EPCR expression by HDMEC cells transfected with either a control siRNA (left) or ADAM10 siRNA (right) and infected with a WT *N*. *meningitidis* strain for 4 hours (red) or left non-infected (green). Grey: isotype control. For the quantification of EPCR expression see supplemental data **S3 Fig**. A Crispr/Cas9-mediated knockdown of ADAM10 expression was performed in hCMEC/D3 as described in the Material and Methods section. (**D**) ADAM10 expression in the resulting cell line (blue) compared to that of the parental cell line (green). **(E)** EPCR expression in the parental hCMEC/D3 cell line (left) or ADAM10-negative-hCMEC/D3 cell line (right) and infected with a WT *N*. *meningitidis* strain for 4 hours (red) or left non-infected (green). Data are representative of 3 independent experiments. (grey: isotype control). For the quantification of EPCR expression see supplemental data **S5 Fig**. **(F)** Soluble EPCR in the supernatant of ADAM10-negative-hCMEC/D3 cells after infection with a WT strain or non-infected (control) cells, assessed by ELISA. Data are mean (+/- SEM) of 3 independent experiments. **(G)** Soluble EPCR in the supernatant of ADAM17-negative or ADAM10-negative hCMEC/D3 cells after 4 hours of treatment with PMA (1 μM). Data are mean (+/- SEM) of 3 independent experiments. *: *p* < 0.0001.

Considering the effect of TAPI-0, a pan-ADAM inhibitor, we next aimed at finding another member of the ADAM family responsible for the EPCR shedding. This family of proteins includes 21 members among which 13 have a predicted or proven proteolytic activity [27]. The sequence comparison of the full proteases or metalloprotease domains showed a segregation of ADAM17 and ADAM10 which are close relatives and are quite distinct from the other members of this family [27,28]. Furthermore, both have been described as sharing several common targets [29]. Therefore, we investigated the role of ADAM10 during meningococcal infection. We first used GI254023X, which is known to specifically inhibit ADAM10 [30]. This drug had no effect on *N*. *meningitidis* adhesion and growth on cells (**S2 Fig**). As shown **Fig 6A**, the EPCR shedding induced by meningococcus infection was significantly reduced in GI254023X-treated HDMEC cells compared to that of DMSO-treated cells. We then used a siRNA approach. The siRNA treatment was associated with a decrease of ~70% of ADAM10 membranous expression (**Fig 6B**). We also verified that ADAM17 expression was not modified in the cells treated with the siRNA against ADAM10 (supplemental data **S3 Fig** and **S4 Fig**). EPCR shedding induced by meningococcal adhesion was dramatically reduced in ADAM10-siRNA-transfected cells compared to that of control-siRNA-transfected cells (**Fig 6C**). To confirm the above results, and since the siRNA knock-down was not complete and was associated with variability in experiments, we engineered an ADAM10-negative-hCMEC/D3 cell line using a Crispr/Cas9 genome edition as described in the material and methods section. The absence of expression of ADAM10 in the resulting cell line was demonstrated by a FACS analysis (**Fig 6D**). The infection of the ADAM10-negative-hCMEC/D3 cells by the WT meningococcal strain was associated with no change of membranous EPCR expression (**Fig 6E**). Moreover, there was no release of soluble forms of EPCR in the supernatant of ADAM10-negative-hCMEC/D3 upon infection (**Fig 6F**). To confirm that ADAM10-negative-hCMEC/D3 cells were still able to shed EPCR after stimulation, we used phorbol 12-myristate 13-acetate (PMA, 1 μM) a known inducer of Protein Kinase C signaling and subsequent ADAM17-mediated EPCR cleavage [26]. As expected PMA treatment had no effect on ADAM17-negative-hCMEC/D3, but efficiently induced an EPCR shedding in ADAM10-negative-hCMEC/D3 (**Fig 6G**).

Altogether the above results demonstrate that meningoccal adhesion on endothelial cells induces an ADAM10-dependent shedding of EPCR.

### *N*. *meningitidis* adhesion on endothelial cells impairs the activation of protein C

**Fig 7:**
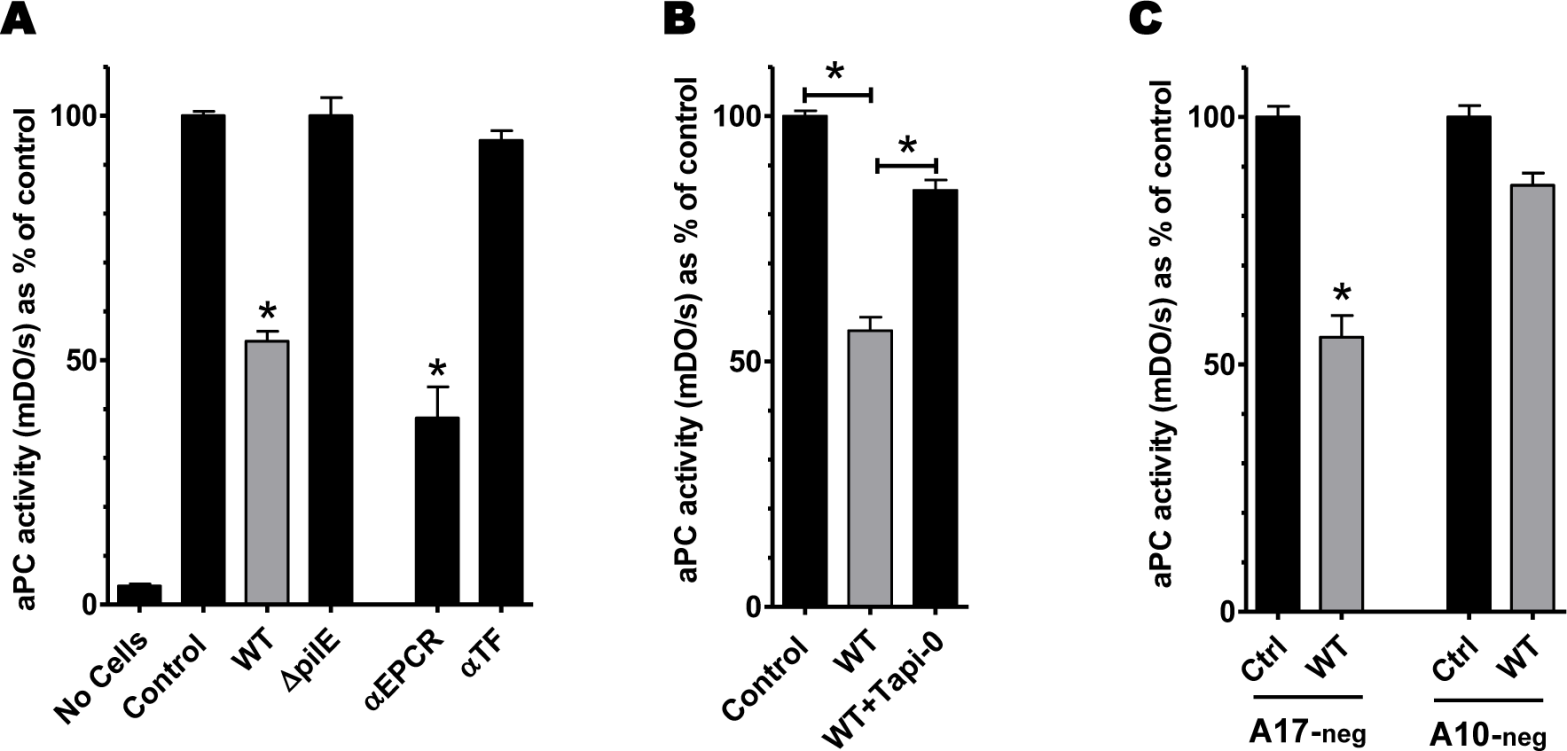
aPC generation by endothelial cells is impaired upon infection by *N*. *meningitidis*. After infection or antibody treatment, endothelial cells were washed and incubated for 30 min with Protein C and thrombin. The amount of aPC generated was determined through its proteolytic activity against a chromogenic substrate. The same experimental procedure was applied to an empty well without cells to assess the spontaneous degradation of the chromogenic substrate (no cells bar). (**A**) HDMEC were infected with either a wild type or Δ*pilE* strain for 4 hours. Cells were also treated for 10 minutes with an antibody blocking EPCR-PC interactions (clone RCR-252, 10 μg/mL) or a control antibody (directed against tissue factor, 10 μg/mL). Data are means (+/-SEM) of % of aPC activity compared to that of non-infected cells from 3 independent experiments. *:p<0.001 compared to control. (**B**) HDMEC cells were infected for 4 hours with a WT strain in the presence or absence of Tapi-0 (25μM). Data are means (+/-SEM) of % of aPC activity compared to that of non-infected cells from 3 independent experiments. (**C**) ADAM17-negative or ADAM10-negative hCMEC/D3 cells were infected for 4 hours with a WT strain. Data are means (+/-SEM) of % of aPC activity compared to that of non-infected cells from 3 independent experiments. *: p<0.0001 compared to control.

PC activation is critical to ensure the control of the coagulation cascade in vivo. PC is activated by endothelial cells following the binding of thrombin on thrombomodulin and PC on EPCR. We demonstrated that meningococcal interaction with endothelial cells induces a profound decrease of EPCR expression through an ADAM10-mediated shedding of this receptor. We then aimed at determining the impact of meningococcal adhesion on the ability of endothelial cells to activate PC. We infected HDMEC for 4 hours with either a WT or a Δ*pilE* non-adhesive isogenic derivative. Following infection, cell monolayers were incubated with thrombin and PC for 30 minutes and the amount of aPC generated was determined using an enzymatic method. As shown **Fig 7A**, infection with the adherent WT strain reduced by 50% the production of aPC, whereas the non-adherent Δ*pilE* strain had no effect. To confirm that this defect in aPC generation was consistent with the loss of EPCR, we incubated cells with a blocking antibody against EPCR that prevents PC interaction with its receptor, limiting the generation of aPC. The reduction of aPC following infection of a monolayer of endothelial cells with the WT strain was comparable to that observed after a complete blocking of PC-EPCR interaction with an antibody (**Fig 7A**).

To demonstrate that this impairment of aPC generation was the consequence of the ADAM10-mediated shedding of EPCR, we first used the pan-ADAM inhibitor TAPI-0. This drug restored the endothelial cells ability to generate aPC when infected with a WT meningococcus (**Fig 7B**). Furthermore, the infection of the ADAM10-negative-hCMEC/D3 cell line by a WT strain had no effect on the generation of aPC, comparatively to that of the ADAM17-negative-hCMEC/D3 cell line (**Fig 7C**).

These data demonstrate that meningococcal adhesion on endothelial cells impairs the activation of PC by inducing an ADAM10-mediated shedding of EPCR.

### The loss of EPCR induced by meningococcal infection suppresses the protective effect of activated protein C on endothelial cells

Beyond its prominent anti-coagulant function, activated Protein C also initiates important endothelial signaling through its receptor. Indeed, aPC fixed on EPCR is able to cleave the Protease Activated Receptor PAR-1 at a non-canonical site. Once cleaved by aPC, the extracellular domain of the PAR-1 receptor acts as a ligand and induces anti-inflammatory and anti-apoptotic responses [31–35]. aPC also initiates a barrier-protective response of the endothelial monolayer, that involves the transactivation of the sphingosine-1-phosphate receptor (S1P), and an increased activity of Rac1 and a decrease activity of RhoA small GTPases. This prevents the formation of actin-myosin stress fibers and reduces the vascular leakage induced by thrombin or pathogens. This protective effect of aPC relies on the presence of EPCR. [34,36–38].

**Fig 8:**
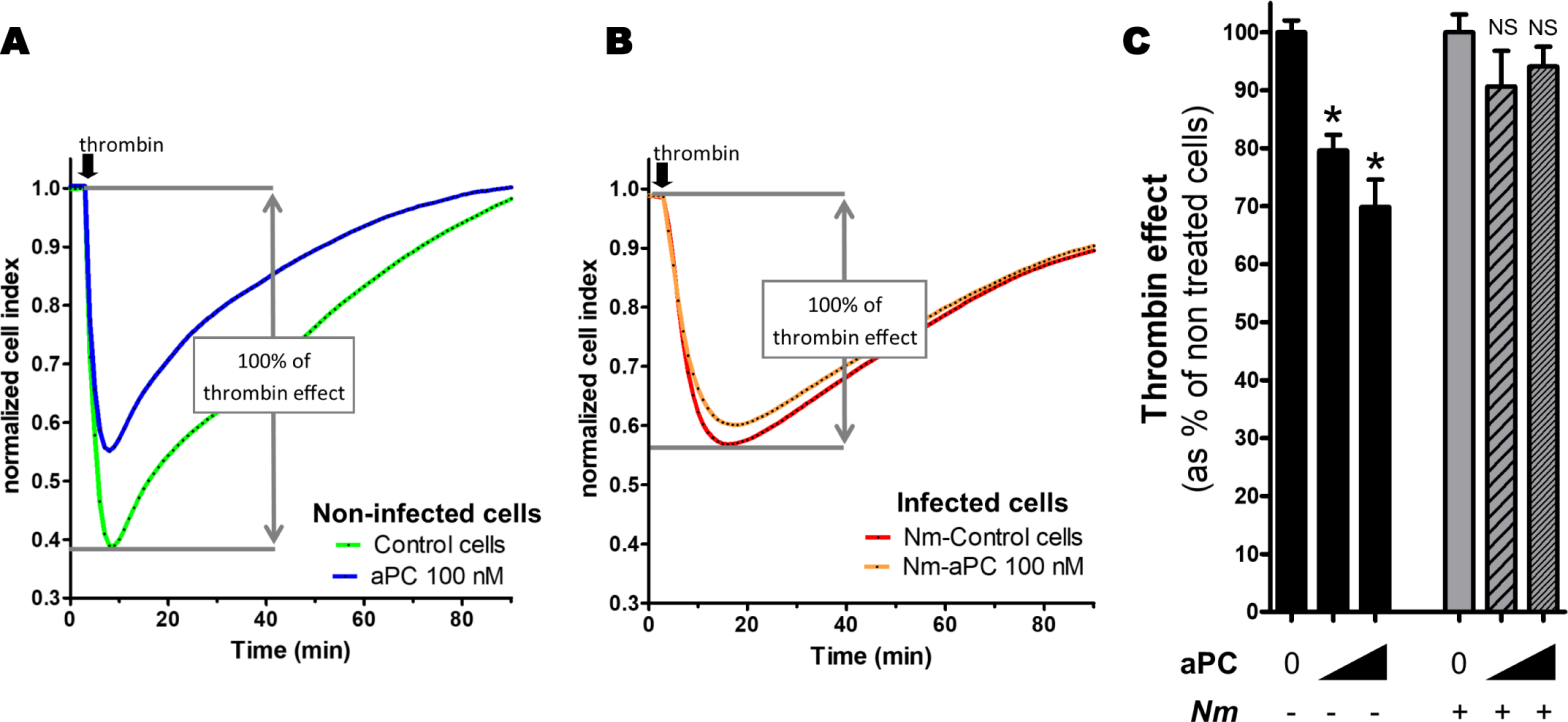
Meningococcal infection suppresses the aPC barrier protective effect. The barrier permeability of HDMEC monolayers was assessed using the iCELLigence system that continuously measures the electrical impedance. Cells were infected with a wild type meningococcus for 4 hours or left non-infected. After the infection, cell media were replaced, and cells were treated with aPC (50 nM or 100 nM) or left untreated. After 2 hours, thrombin (1 nM) was added and electrical impedance (Cell Index) was measured every minutes. (**A**) Representative data showing the effect of thrombin on monolayers impedance when HDMEC cells were treated (blue) or not (green) with aPC (100 nM). (**B**) Representative data showing the effect of thrombin on monolayers impedance when HDMEC cells were treated (orange) or not (red) with aPC (100 nM). (**C**) Loss of CI induced by thrombin expressed as % of non-treated cells. Data are mean (+/-SEM) from three independent experiments. For each condition (non-infected/infected) an analysis of variance (ANOVA) was done followed by multiple comparisons by t-tests with a correction of Bonferonni. *: *p* <0.01 compared to non-treated cells. NS: non significant (*p*>0.05).

To investigate the effect of the meningococcal-induced EPCR shedding on the barrier-protective signaling of aPC, we used the iCELLigence System that continuously monitors the electrical impedance across the endothelial monolayer and enables a real-time analysis of the barrier function and integrity. Confluent monolayers of HDMEC cells grown on ACEA Plates L8 were infected with *N*. *meningitidis* or left uninfected. After 4 hours, the cell culture medium was replaced by fresh medium and activated protein C was added (50 and 100 nM) for 2 hours. Thrombin (1 nM) was added to induce the barrier disruption. As expected, in non-infected cells, aPC provided a protection against thrombin (**Fig 8A** and **8C**). On the other hand, meningococci-infected monolayers were not protected against thrombin-induced disruption (**Fig 8B** and **8C**). These data demonstrate that meningococcal-induced EPCR shedding abrogates the aPC/EPCR/PAR-1 protective signaling.

## Discussion

The activation of coagulation is a common response to pathogens invasion. However, a particular pro-thrombotic activity is observed during *N*. *meningitidis* infections. Indeed, most meningococal infections are associated with cutaneous purpuric lesions which are the clinical consequence of dermal microvessels thrombosis. In severe forms, the latter extend and evolve toward a dreadful *purpura fulminans* syndrome with extensive necrosis.

Here, we demonstrate that meningococcal adhesion on endothelial cells induces a rapid and intense proteolytic cleavage of the EPCR from the endothelial membrane. This result is consistent with previous studies showing a reduced EPCR endothelial staining in purpuric lesions [15,39]. In addition, we demonstrate that this cleavage has functional consequences by impairing the generation of aPC by endothelial cells. *In vivo* studies clearly demonstrate that EPCR is key for the generation of sufficient aPC and for the subsequent control of the coagulation cascade after a pro-thrombotic trigger. PC activation following a thrombin challenge is reduced by 95% in an EPCR deficient mice [40] and by 92% in mice expressing a mutated EPCR unable to bind PC [14]. Similarly, in baboons, the blockade of PC-EPCR interaction with an antibody results in a 87% decrease of aPC generation following the infusion of thrombin [12]. As a consequence, in all the above-referenced animals models, any thrombotic challenge–such as a Gram negative systemic infection—induces the extensive thrombosis of several organs and death [11,12,14,40,41]. As severe meningococcemia is associated with high levels of circulating endotoxin and pro-inflammatory cytokines that trigger an important activation of the coagulation cascade, the decrease of aPC induced by the bacterial colonization of skin microvessels is a likely explanation of cutaneous purpura and purpura fulminans.

Beyond the decrease of aPC generation, the loss of EPCR is also highly detrimental. Indeed, EPCR is key to the non-anticoagulant properties of aPC. aPC fixed on endothelial EPCR is able to cleave the Protease Activated Receptor PAR-1 at a non-canonical site, initiating barrier-protective, cytoprotective and anti-inflammatory signalings [31–38]. We demonstrate here that meningococcal adhesion on endothelial cells and the subsequent EPCR shedding inhibit the barrier protective effect of the aPC/EPCR/PAR-1 signaling. As this signaling counteracts endothelial barrier disruption during sepsis, it is tempting to speculate that its impairment could participate in refractory septic shock, capillary leakage and blood-brain barrier crossing that are common during meningococcemia.

Thus, it arises from our data that meningococcus affects both the anticoagulant properties of aPC by limiting its activation, but also its broad barrier-protective, cytoprotective and anti-inflammatory properties by preventing its interaction with EPCR, cleaved upon meningococcal adhesion.

We provide clear evidences that EPCR is cleaved by ADAM10 upon meningococcal adhesion, with no involvement of ADAM17. To date, only ADAM17 was known as shedding EPCR [26]. However, it is now clear that ADAM17 and ADAM10 share common targets and that one protease can be activated independently from the other, depending on the initial trigger [29,42]. Our work gives a new insight into EPCR biology, and the respective roles of ADAM17 and ADAM10 in pathological contexts involving EPCR remains to be clarified. It is also interesting to point out that ADAM10 has several other targets whose shedding may contribute to *N*. *meningitidis* pathogenesis. This protease is notably able to cleave endothelial VE-Cadherin, a key component of endothelial intercellular junctions and a critical determinant of barrier integrity [43–45]. Interestingly, *Staphylococcus aureus* alpha-hemolysin (Hla) has been shown to bind to and to activate ADAM10 [46]. This activation induces VE-Cadherin cleavage responsible for a capillary leakage in a mouse model of Hla intoxination [47]. Therefore, it is likely that ADAM10 activation upon *N*. *meningitidis* vascular colonization induces VE-cadherin cleavage and participates to the capillary leakage and eventually to the crossing of the blood-brain barrier by the bacteria.

Type IV pilus is the critical determinant of the meningococcal interaction with endothelial cells. It is essential to the initial adhesion on cells and to subsequent vascular colonization [21]. We demonstrated that pilus interaction with endothelial cells is also required for ADAM10 activation and EPCR shedding. Our data bring out a new cellular response triggered by meningococcal pilus. ADAM10 has a prominent role in several physiological and pathological pathways such as Notch signaling, embryonic development, tumor resistance to anticancerous agents or protection against Alzeihemer’s disease [48]. As such, its has been extensively studied but many questions remain unanswered to date. The mechanism by which it switches form an inactive state to an active state by a change of conformation has been described very recently [49]. But the precise cellular mechanisms that triggers this changing of conformation are still not understood. Therefore, further work is needed to decipher the mechanism by which ADAM10 is activated following pilus-mediated adhesion of *N*. *meningitidis*.

This work, by identifying for the first time a specific dysregulation of the coagulation induced by *N*. *meningitidis*, opens therapeutics perspectives. Indeed, human recombinant aPC was a licensed drug, approved in 2001 with a broad indication of severe septic shocks. It was intended to improve survival due to the multiple effects of aPC [50]. After a promising preliminary study, the drug failed to confirm efficiency in several subsequent controlled trials [51,52] and was sometimes associated with an increased risk of serious bleeding. The drug was subsequently withdrawn from the market in 2011 but the real benefit of the drug is still a matter of debate [52–55]. However, it should be pointed out that recombinant aPC has never been evaluated in the specific context of meningococcal purpura fulminans, in contrast to extensive data with the non-activated zymogen [15,56–65]. Furthermore, the clinical endpoint of the aPC trials was sepsis-induced mortality and not thrombosis sequelae. Our data raises the question whether patients suffering from meningococcal invasive infection could benefit from an aPC treatment to prevent or limit thrombosis and thrombosis sequelae.

In summary, *N*. *meningitis* endothelial colonization mediated by type IV pili triggers ADAM10 activation and subsequent EPCR shedding that impairs the generation of aPC.All three events could participate—and synergize—in meningococcal induced vascular injury, capillary leakage, fluid loss, and the bacterial crossing of the blood-brain barrier. Despite active antimicrobials and continuous improvements in intensive care, meningococcus purpura fulminans is still associated with a poor outcome and severe thrombosis sequelae such as amputations. Therefore, our work set up a basis for the development of new therapeutics based on ADAM10 inhibition and/or aPC supplementation alongside with antibiotics and traditional intensive care.

## Materials and methods

### Cell lines

Human Dermal Microvessels Endothelial Cells (HDMEC) were purchased from Promocell and grown in Endothelial Cell Medium MV (Promocell) containing 5% Fetal Calf Serum (FCS) and endothelial cell growth supplements at 37°C/5%CO2. Human Cerebral Microvessels Endothelial Cells (hCMEC/D3) were a gift from P.O. Couraud at Institut Cochin, Paris, France, and were grown in Endothelial Cell Basal Medium-2 (Lonza) supplemented with 5% of FCS, 1.4 μM hydrocortisone (Lonza), 5 μg/ml ascorbic acid (Lonza), 1 ng/ml b-FGF (Lonza), at 37°C in 5% CO2.

### Bacterial strains

A piliated capsulated serogroup C *N*. *meningitidis* clinical strain 2c4.3 was used in this study [66]. An isogenic non-piliated Δ*pilE* derivative of strain 2c4.3 was previously described [66,67] In order to test the specific role of T4P we used a previously described isogenic derivative of strain 2c4.3 which is non piliated (Δ*pilE)*, and non capsulated (Δ*siaD)*[23]. This strain is unable of pilus-mediated adhesion but can interact with cells via Opa proteins, a family of outermembrane proteins that mediate adhesion through their interaction with CEACAMs (Carcino Embryonic Antigen-related Cell Adhesion Molecule) receptors [68]. It should be pointed out that this interaction between Opa and CEACAMs occurs only if bacteria are non-capsulated. As the Opa-mediated adhesion on endothelial cells is weaker than that induced by T4P, cells were infected with a multiplicity of infection (MOI) twenty times higher than that used when a WT piliated strain was used for infection. Under such conditions, meningococcal interaction with endothelial cells is similar to that observed with the conditions used when infecting with a WT strain.

Strains were grown on gonococcal-broth agar with Kellogg’s supplements at 37°C/5%CO_2_.

### Infection of endothelial cells cultures

Before infection bacteria were grown in cell medium for 2 hours to reach the growth exponential phase. Cells were infected with a Multiplicity Of Infection (MOI) of 25 bacteria per cell unless specified. When needed, Tapi-0 (25μM, Merck Millipore) or GI254023X (1 μM, Bio-Techne) were added 1h before infection.

### Flow cytometry

After infection, cells were washed twice in Phosphate-Buffered-Saline (PBS), trypsinized, fixed in 4% PFA for 10 minutes, washed twice in PBS and kept at 4°C until staining. Cells were stained for 1 hour with the appropriate primary antibody at 4°C in PBS/Bovine Serum Albumine (BSA) 1% and washed twice in PBS-BSA before FACS analysis. The following antibodies were used: EPCR (clone RCR-252, PE-conjugated, BD Biosciences), Thrombomodulin (clone 1A4, PE-conjugated, BD Bioscience), ADAM17 (clone 111633, PE-conjugated, R&D Systems), and ADAM10 (clone 163003, PE-conjugated, R&D Systems). Isotype controls were chosen accordingly. Data were acquired using a BD LSR Fortessa instrument (BD Biosciences) and analyzed using the FlowJo Software version 10. A minimum of 5 000 cells were acquired for each experiment.

### Immunofluorescence assays

For immunofuorescence assays, cells were grown on glass coverslips coated with 5 μg/cm2 of rat tail collagen type I (Corning) until confluence. After infection cells were fixed in 4% PFA for 5 minutes, washed twice in PBS, and incubated 15 minutes in PBS/BSA 1%. EPCR staining was performed first with no cell permeabilization (polyclonal goat antibody from R&D Systems). Cells were then washed twice in PBS and permeabilized with Triton X-100 (0.1% Sigma). VE-Cadherin staining was performed after permeabilization (polyclonal rabbit antibody from eBioscience). After staining cells were washed twice in PBS and incubated with secondary antibodies (Alexa-fluor conjugated, invitrogen) and DAPI (Invitrogen). After additional washings, coverslips were mounted in mowiol (Biovalley). Images acquisition was performed on a laser-scanning confocal microscope (Leica TCS SP5). Images were collected and processed using the Leica Application Suite AF lite (Leica) software.

### Soluble EPCR ELISA

After infection, the supernatants were collected and centrifuged at 4000 rpm for 10 minutes at 4°C. Soluble EPCR was dosed using an ELISA commercial kit (R&D Systems). For each experiment, a standard curve was generated using the highly purified human EPCR provided with the kit, from 0.313 ng/mL to 20 ng/mL following the manufacturer’s instructions. Each point was dosed in duplicate. Data were analysed using a 4-Parameters Logistic Regression and the online software www.elisaanalysis.com. The limit of detection and the limit of linearity using this kit is then of 0.313 ng/mL. All the cell supernatants had a sEPCR concentration superior to 0.313 ng/mL. The specificity of the capture antibody from the kit was tested using a whole cell lysate of Human Dermal Microvessels Endothelial Cells (10 μg/lane) and a Western-Blot analysis (antibody concentration: 0.5 μg/mL). The capture antibody detected a unique band of approximately 48 kDa which is the molecular weight of the glycosylated EPCR.

### siRNA experiments

Ambion Silencer Select siRNA targeting ADAM17, ADAM10 or control siRNA were purchased from ThermoFisher Scientific (references S13720, S1006 and 4390843 respectively). HDMEC cells were transfected with siRNA (2 μM) using AMAXA transfection system (Lonza), Human Umbilical Vein Endothelial Cells (HUVEC) transfection kit (Lonza) and program HUVEC-OLD, according to manufacturer’s protocol. Cells were infected 3 days after transfection.

### Clustered Regularly Interspaced Short Palindromic Repeats (CRISPR) / Cas9 genome edition

CRISPR/Cas9-mediated genome editions were conducted using Dr. Feng Zhang Lab CRISPR tools and protocols (http://www.genome-engineering.org and Cong *et al* [69]). The following sequences were used as target and cloned into pSpCas9(BB)-2A-GFP (PX458) plasmid, a gift from Feng Zhang (Addgene #48138): ADAM17 (GTCGCGGCGCCAGCACGAA), ADAM10 (CGTCTAGATTTCCATGCCCA). The efficiency of each construct to cleave target sequences was first analyzed in HEK cells by a T7 Endonuclease I assay using the Gene Art Genomic Cleavage Detection kit (ThermoFisher Scientific) according to the manufacturer’s protocol. hCMEC/D3 cells were transfected with the constructed plasmids (1 μg/100μL) using AMAXA transfection system (Lonza), HUVEC transfection kit (Lonza) and program HUVEC-OLD. The day after, cells were trypsinized and suspended in cold HBSS/FCS (2%). GFP-positive cells were selected using an Aria III cell sorter (BD Bioscience). The whole GFP-positive populations were grown for two weeks and ADAM17 or ADAM10 negative cells were then selected using the Aria III cell sorter. The whole negative populations were grown, amplified and used for the experiments. The absence of ADAM17 or ADAM10 expression was also verified during each experiment.

### Protein C activation assay

The protocol was adapted from a previously published paper [10]. Cells were grown in 1.9 cm^2^ wells until confluence. After infection, the monolayer was washed twice with Hank’s Balanced Salt Solution (HBSS) supplemented with 0.1%BSA, 3 mM CaCl_2_ and 0.6 mM MgCl_2_ before a 5-minutes incubation with 200 nM Protein C (Enzyme Research Laboratories) in a total volume of 200 μL of the same buffer at 37°c under gentle agitation. Activated thrombin (10 nM, Diagnostica Stago) was added to initiate the reaction. After 30 min, 150 μL of the supernatant was collected and antithrombin (1.8 μM, Aclotine from Laboratoire de Fractionnement et des Biotechnologies—LFB, Courtaboeuf, France) and heparin (90 mU/mL, from Sanofi-Aventis, France) were added to stop the reaction. The activity of aPC was determined using 200 μM of CS-21-66 (Hyphen Biomed) as chromogenic substrate and a Mithras LB940 plaque reader (Berthold Technologies). All aPC determinations were done in duplicate.

### Endothelial barrier permeability assay

Endothelial cell permeability was measured in real time using the iCELLigence System (Acea Biosciences) that determines the changes in transendothelial resistance by electric-substrate impedance sensing (ECIS). HDMEC (50 000 / well) were seeded in E-Plate L8 wells containing gold electrodes (Acea Biosciences). The Cell Index (CI) that reflects the ECIS was monitored every 20 minutes for 48 hours until complete stabilization. Endothelial cells were infected for 4 hours or left non-infected. After infection, the cell medium was removed to get rid of soluble EPCR and replaced by fresh medium without heparin. Cells were then treated for 2 hours with Activated Protein C (50 nM or 100 nM, Lilly) or left untreated. After 2 hours, the CI were normalized and recorded every minute. Thrombin (1 nM, Diagnostica Stago) was added in each well. For comparisons, the maximal loss of CI induced by thrombin in cells not treated by aPC was set to 100%.

## Supporting information

S1 Fig Quantification of EPCR expression in HDMEC cells. Cells were infected for 4 hours with a wild-type (WT) meningococcus strain or the indicated mutant or left non-infected. When a drug was used, the non-infected cells were also treated in the same conditions. After infection, EPCR expression was assessed by a FACS analysis. For each experiment, the Mean Fluorescence Intensity (MFI) of the non-infected cells was set to 100%. Data are mean (+/-SEM) of MFI from at least 3 independent experiments. **: *p* <0.0001. *: *p*< 0.05 (one-sample *t-test* comparing the mean to the hypothetical value of 100).
(TIF)

S2 Fig Cell-adherent meningococcus on HDMEC cells treated with ADAMs inhibitors. HDMEC cells were grown in 1.8 cm^2^ wells until confluence. Cells were treated for 1 hour with Tapi-0 (25 μM), GI254023X (1 μM) or DMSO and were subsequently infected with a WT N. meningitidis strain for 4 hours in the presence of the drug. After infection, cells were washed 5 times in endothelial cell medium to remove non-adherent bacteria and scraped in 1 mL of medium. Data are mean (+/- SEM) of colony forming units (cfu) / well of cell-adherent bacteria from 3 independent experiments.
(TIF)

S3 Fig Quantification of EPCR, ADAM17 and ADAM10 expression in HDMEC cells. HDMEC cells were treated with siRNA against ADAM17 or ADAM10 or a control siRNA.
(**A**). ADAM17 expression was assessed using a FACS analysis. The MFI of siRNA-control treated cells was set to 100%. Data are mean (+/-SEM) of MFI from 3 independent experiments. *: p<0.01. (one-sample *t-test* comparing the mean to the hypothetical value of 100).
(**B**) siRNA-treated cells were infected with the WT strain of *N*. *meningitidis* for 4 hours or left uninfected. After infection, EPCR expression was assessed by a FACS analysis. For each experiment, the Mean Fluorescence Intensity (MFI) of the non-infected cells was set to 100%. Data are mean (+/-SEM) of MFI from 3 independent experiments. **: *p* < 0.01; *: *p* < 0.05; NS: non-significant (one-sample *t-test* comparing the mean to the hypothetical value of 100).
(**C**) ADAM10 expression was assessed using a FACS analysis. The MFI of siRNA-control treated cells was set to 100%. Data are mean (+/-SEM) of MFI from 3 independent experiments. *: p<0.01. (one-sample *t-test* comparing the mean to the hypothetical value of 100).
(TIF)

S4 Fig ADAM17 expression in HDMEC cells treated with a siRNA against ADAM10. HDMEC cells were treated with a siRNA against ADAM10 (blue) or a control siRNA (green, tinted) and ADAM17 expression was assessed using a FACS analysis. A representative result is shown. For quantification see **S3 Fig**.
(TIF)

S5 Fig Quantification of EPCR expression in hCMEC/D3 cells and ADAM17 or ADAM10-negative derivatives. A Crispr/Cas9 technology was used to engineer ADAM17 or ADAM10 negatives hCMEC/D3 cell lines. Cells were infected with the WT meningococcus strain for 4 hours or left uninfected. After infection, EPCR expression was assessed by a FACS analysis. For each experiment, the Mean Fluorescence Intensity (MFI) of the non-infected cells was set to 100%. Data are mean (+/-SEM) of MFI from 3 independent experiments. *: *p* < 0.01; NS: non-significant (one-sample *t-test* comparing the mean to the hypothetical value of 100).
(TIF)
