# Supplementary figures and images for: An ADAM-10 dependent EPCR shedding links meningococcal interaction with endothelial cells to purpura fulminans
Source: PLoS Pathog. 2018 Apr 9;14(4):e1006981. doi: 10.1371/journal.ppat.1006981 (PMC5908201; doi:10.1371/journal.ppat.1006981)

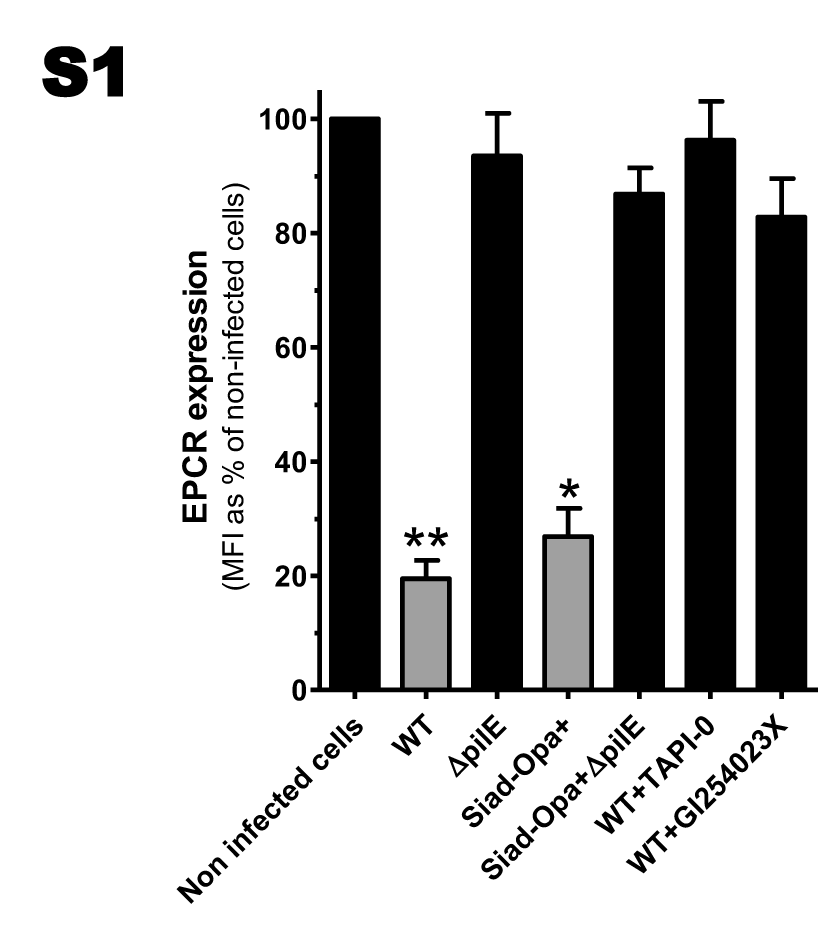

Supplement: S1 Fig — Cells were infected for 4 hours with a wild-type (WT) meningococcus strain or the indicated mutant or left non-infected. When a drug was used, the non-infected cells were also treated in the same conditions. After infection, EPCR expression was assessed by a FACS analysis. For each experiment, the Mean Fluorescence Intensity (MFI) of the non-infected cells was set to 100%. Data are mean (+/-SEM) of MFI from at least 3 independent experiments. **: p <0.0001. *: p< 0.05 (one-sample t-test comparing the mean to the hypothetical value of 100). (TIF) [file ppat.1006981.s001.tif]

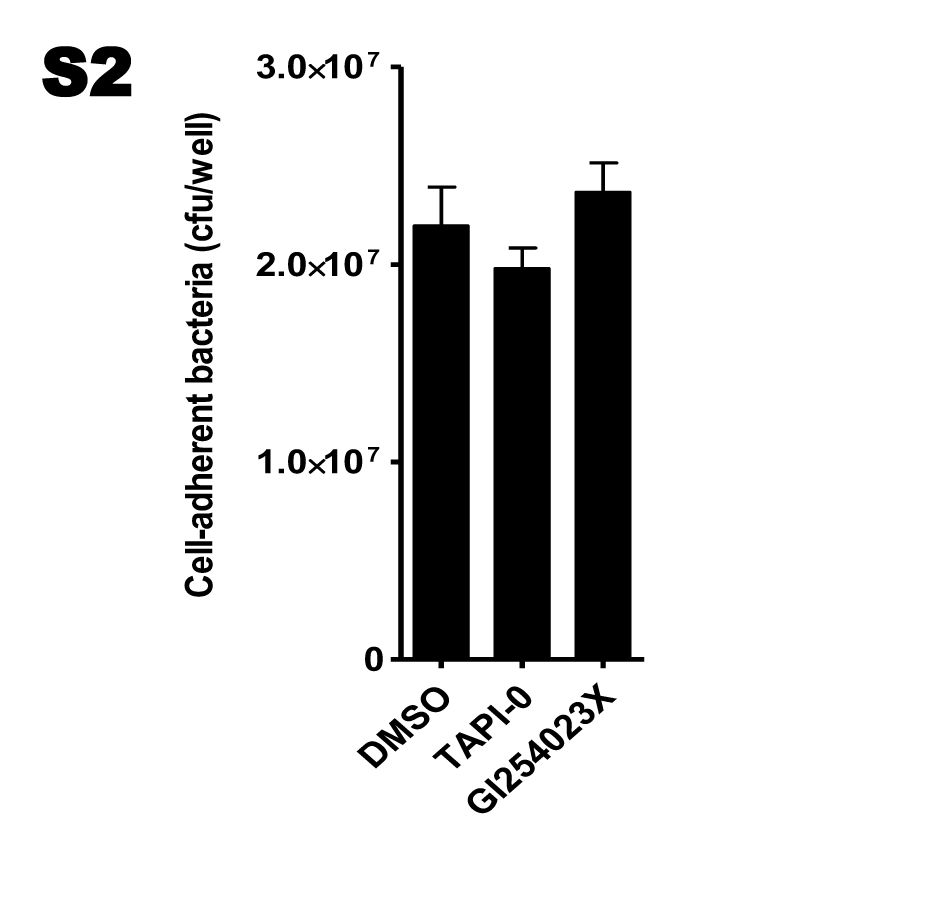

Supplement: S2 Fig — HDMEC cells were grown in 1.8 cm2 wells until confluence. Cells were treated for 1 hour with Tapi-0 (25 μM), GI254023X (1 μM) or DMSO and were subsequently infected with a WT N. meningitidis strain for 4 hours in the presence of the drug. After infection, cells were washed 5 times in endothelial cell medium to remove non-adherent bacteria and scraped in 1 mL of medium. Data are mean (+/- SEM) of colony forming units (cfu) / well of cell-adherent bacteria from 3 independent experiments. (TIF) [file ppat.1006981.s002.tif]

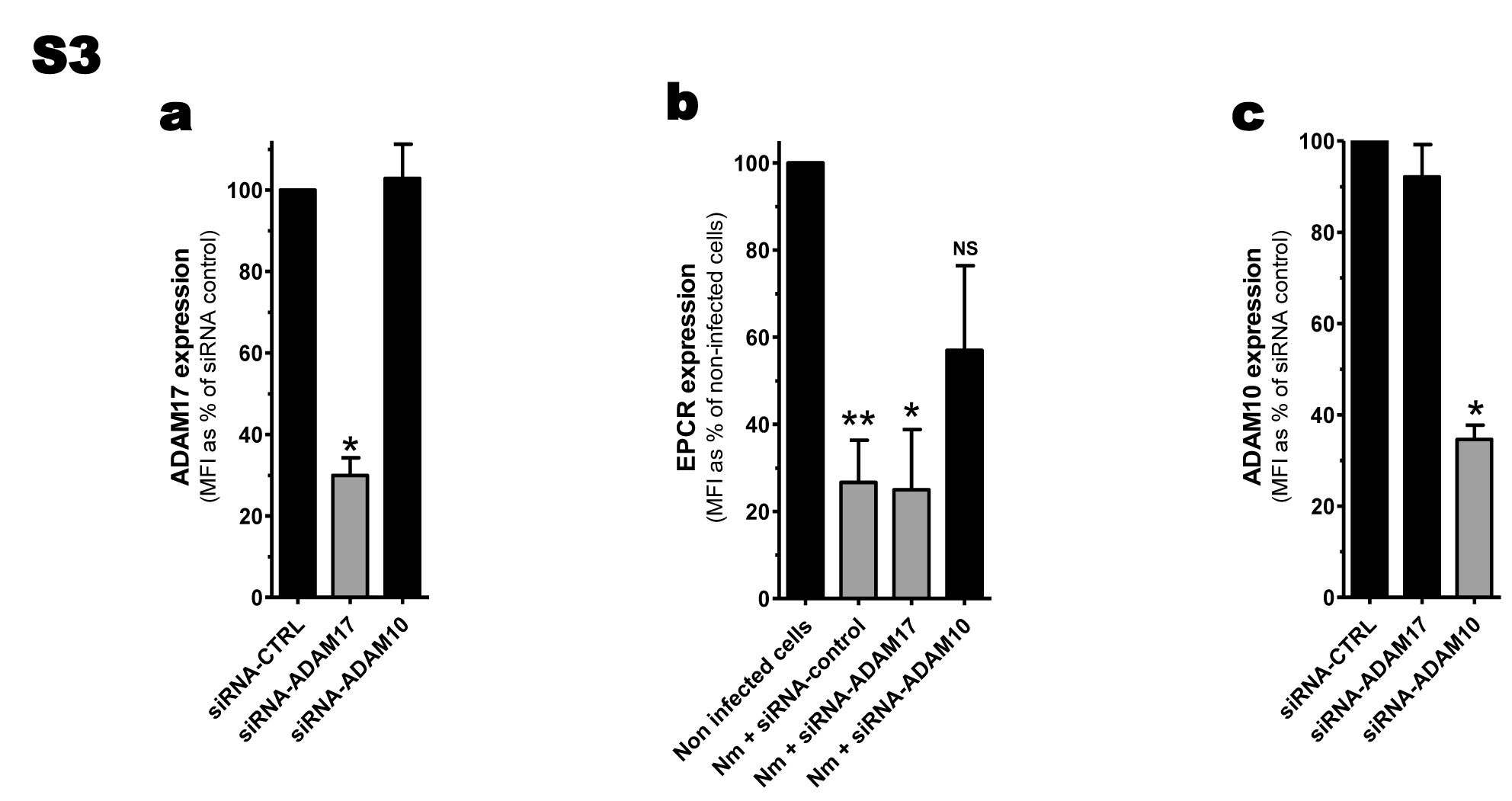

Supplement: S3 Fig — HDMEC cells were treated with siRNA against ADAM17 or ADAM10 or a control siRNA. (A). ADAM17 expression was assessed using a FACS analysis. The MFI of siRNA-control treated cells was set to 100%. Data are mean (+/-SEM) of MFI from 3 independent experiments. *: p<0.01. (one-sample t-test comparing the mean to the hypothetical value of 100). (B) siRNA-treated cells were infected with the WT strain of N. meningitidis for 4 hours or left uninfected. After infection, EPCR expression was assessed by a FACS analysis. For each experiment, the Mean Fluorescence Intensity (MFI) of the non-infected cells was set to 100%. Data are mean (+/-SEM) of MFI from 3 independent experiments. **: p < 0.01; *: p < 0.05; NS: non-significant (one-sample t-test comparing the mean to the hypothetical value of 100). (C) ADAM10 expression was assessed using a FACS analysis. The MFI of siRNA-control treated cells was set to 100%. Data are mean (+/-SEM) of MFI from 3 independent experiments. *: p<0.01. (one-sample t-test comparing the mean to the hypothetical value of 100). (TIF) [file ppat.1006981.s003.tif]

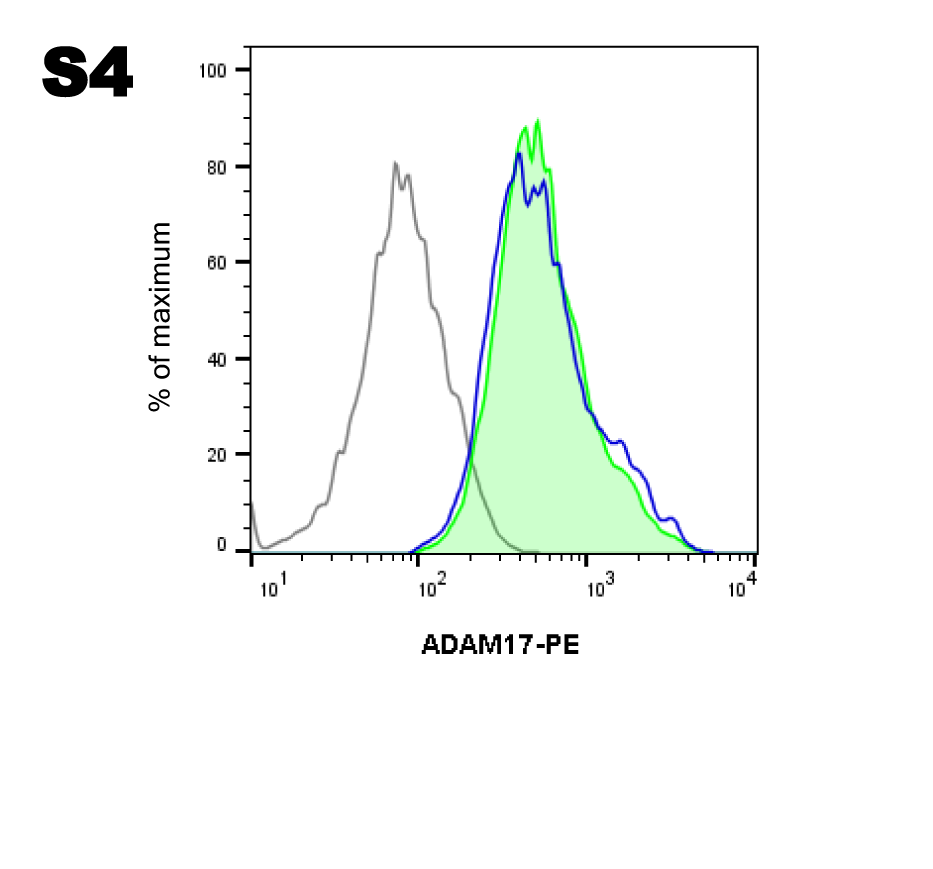

Supplement: S4 Fig — HDMEC cells were treated with a siRNA against ADAM10 (blue) or a control siRNA (green, tinted) and ADAM17 expression was assessed using a FACS analysis. A representative result is shown. For quantification see S3 Fig. (TIF) [file ppat.1006981.s004.tif]

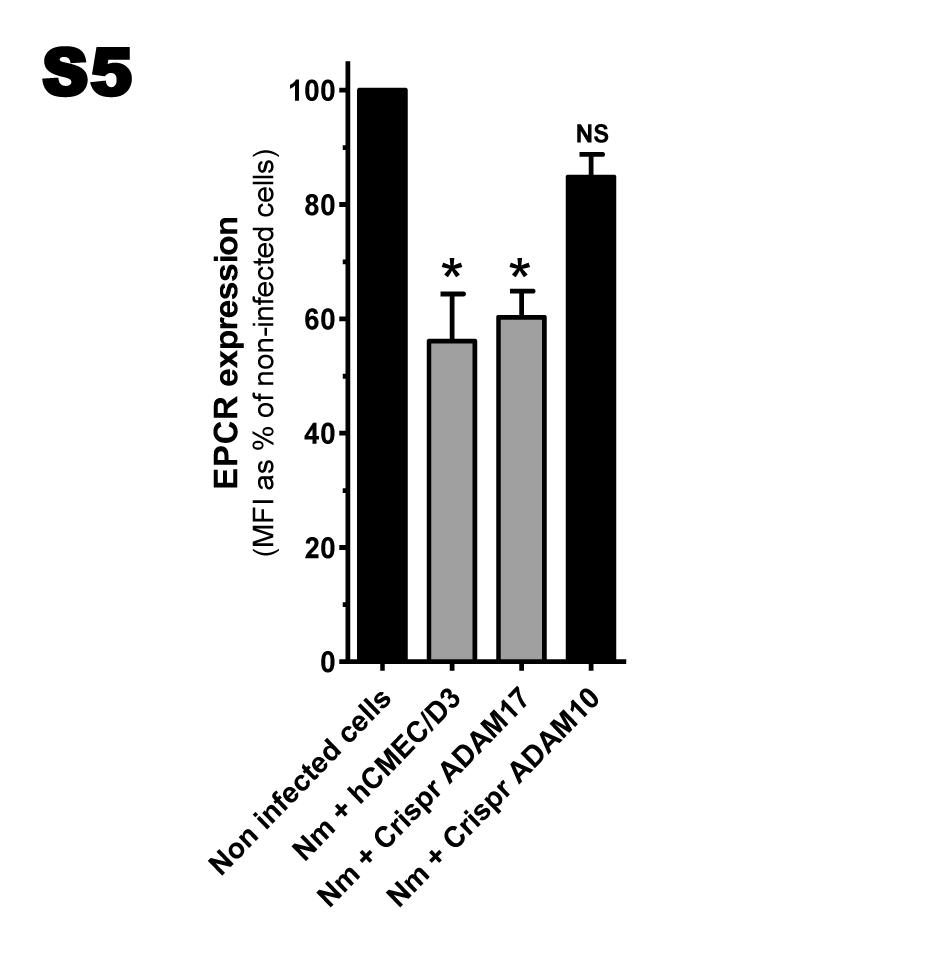

Supplement: S5 Fig — A Crispr/Cas9 technology was used to engineer ADAM17 or ADAM10 negatives hCMEC/D3 cell lines. Cells were infected with the WT meningococcus strain for 4 hours or left uninfected. After infection, EPCR expression was assessed by a FACS analysis. For each experiment, the Mean Fluorescence Intensity (MFI) of the non-infected cells was set to 100%. Data are mean (+/-SEM) of MFI from 3 independent experiments. *: p < 0.01; NS: non-significant (one-sample t-test comparing the mean to the hypothetical value of 100). (TIF) [file ppat.1006981.s005.tif]
